# Remote ischemic preconditioning to reduce contrast-induced nephropathy: study protocol for a randomized controlled trial

**DOI:** 10.1186/1745-6215-15-119

**Published:** 2014-04-11

**Authors:** Thomas B Sterenborg, Theo P Menting, Yvonne de Waal, Rogier Donders, Kimberley E Wever, M Susan Lemson, Daan JA van der Vliet, Jack F Wetzels, Leo J SchultzeKool, Michiel C Warlé

**Affiliations:** 1 Department of Surgery, Division of Vascular- and Transplant Surgery, Radboud University Nijmegen Medical Centre, Geert Grooteplein-Zuid 10, Nijmegen, GA 6525, the Netherlands; 2 Department of Nephrology, Nijmegen, the Netherlands; 3 Department of Epidemiology Biostatistics and HTA, Nijmegen, the Netherlands; 4 Department of Radiology, Radboud University Nijmegen Medical Centre, Nijmegen, the Netherlands; 5 Department of Surgery, Division of Vascular Surgery, Slingeland Hospital, Doetinchem, the Netherlands

**Keywords:** Contrast-induced nephropathy, Remote ischemic preconditioning, Acute kidney injury, Pre- and posthydration, Randomized controlled trial

## Abstract

**Background:**

Despite the increasing use of pre- and posthydration protocols and low-osmolar instead of high-osmolar iodine-containing contrast media, the incidence of contrast-induced nephropathy (CIN) is still significant. There is evidence that contrast media cause ischemia-reperfusion injury of the medulla. Remote ischemic preconditioning (RIPC) is a non-invasive, safe, and low-cost method to reduce ischemia-reperfusion injury.

**Methods:**

The RIPCIN study is a multicenter, single-blinded, randomized controlled trial in which 76 patients at risk of CIN will receive standard hydration combined with RIPC or hydration with sham preconditioning. RIPC will be applied by four cycles of 5 min ischemia and 5 min reperfusion of the forearm by inflating a blood pressure cuff at 50 mmHg above the actual systolic pressure. The primary outcome measure will be the change in serum creatinine from baseline to 48 to 72 h after contrast administration.

**Discussion:**

A recent pilot study reported that RIPC reduced the incidence of CIN after coronary angioplasty. The unusual high incidence of CIN in this study is of concern and limits its generalizability. Therefore, we propose a randomized controlled trial to study whether RIPC reduces contrast-induced kidney injury in patients at risk for CIN according to the Dutch guidelines.

**Trial registration:**

Current Controlled Trials ISRCTN76496973

## Background

Iodine-containing contrast media are often used for diagnostic and therapeutic procedures and their use is the leading cause of hospital-acquired acute kidney injury [1]. Prospective studies demonstrate that contrast media are responsible for approximately 15% of acute kidney injury cases [2,3]. Despite the increasing use of pre- and posthydration protocols and low-osmolar instead of high-osmolar iodine-containing contrast media, the incidence of contrast-induced acute kidney injury is still significant [4,5]. This so called contrast-induced nephropathy (CIN) is defined as an absolute rise of ≥0.5 mg/dL and/or a relative increase of ≥25% in serum creatinine compared to baseline within 48 to 72 h after contrast administration without an alternative cause of kidney injury [6]. CIN is strongly associated with morbidity and mortality [7,8]. In patients with CIN, 8% need dialysis treatment and between 22% and 34% die during the index hospitalization [3,9-11]. In accordance with international guidelines, all patients who receive iodine-containing contrast are screened for risk factors of CIN, including measures of renal function (estimated glomerular filtration rate, based upon the MDRD formula) [12-15]. High-risk patients receive pre- and posthydration by saline solution infusion for 4 to 12 h. Furthermore, 48 to 72 h after contrast administration, serum creatinine should be measured [16]. Despite the identification of high-risk patients and the use of hydration protocols, the incidence of CIN still varies between 2% and 13% [17-20]. The exact mechanism underlying CIN remains to be elucidated. There is evidence to suggest that contrast media have direct toxic effects on the tubular cells resulting in altered mitochondrial function and apoptosis [21]. Moreover, ischemia-reperfusion injury of the medulla has been shown to play an important role [22]. The outer part of the medulla has an area with a high oxygen demand and is located at a distance from the vasa recta which supplies the medulla of blood. Contrast-induced vasoconstriction of the vasa recta induces ischemia-reperfusion injury of the medulla which contributes significantly to the pathophysiology of CIN. Remote ischemic preconditioning (RIPC) is a short and harmless discontinuation of blood supply to particular organs or tissue, followed by reperfusion [23,24]. A preconditioning stimulus is applied before the onset of prolonged ischemia. In animal models it has been found to reduce ischemia-reperfusion injury of the kidney [25]. Although the precise mechanism of RIPC remains unknown, two major pathways may play a pivotal role: the humoral and neurogenic pathways. Both are thought to induce various kinase cascades and eventually prevent opening of the mitochondrial permeability transition pore in the target organ, thereby reducing cell death [26]. A retrospective cohort study by Whittaker et al. indicated that multiple balloon inflations during coronary angioplasty (as a remote stimulus) might reduce CIN [27]. Furthermore, a recent pilot study by Er et al. showed that RIPC reduced CIN in high-risk patients undergoing elective coronary angiography [28]. However, there was an unusually high incidence of CIN (40%) in the control group. The question arises whether protection by RIPC, as an adjunct to standard preventive measures (that is, hydration and discontinuation of nephrotoxic drugs), also holds for patients with a lower risk of CIN. As generalizability of the results by Er et al. is confined to a selected group of patients with an unusual high risk of CIN, we propose a randomized controlled trial to study whether RIPC reduces contrast-induced kidney injury in patients at risk of CIN according to the Dutch guideline [14].

## Methods/Design

A multicenter, single-blinded, randomized controlled trial will be performed at the Radboud University Nijmegen Medical Centre and Slingeland Hospital Doetinchem. Inclusion will be performed by the physician researcher after written informed consent.

### Study population

**Figure 1:**
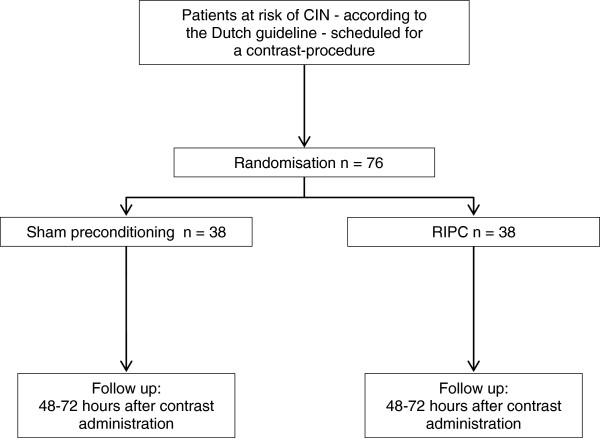
**Study flow chart.** Legend: n.a.

A total of 76 patients will be randomized. Sealed envelopes are used to randomly assign consecutive patients in a 1:1 ratio to receive either sham preconditioning or RIPC (Figure 1). The study population consists of patients at risk of CIN according to criteria adopted from the Dutch guidelines: (1) eGFR <45 mL/min/1.73 m^2^; (2) eGFR <60 mL/min/1.73 m^2^; (3) eGFR <60 mL/min/1.73 m^2^ and two additional risk factors (that is, peripheral vascular disease, heart failure, >75 years of age, anemia, dehydration, use of diuretics and/or NSAIDs). Patients undergoing contrast procedures for diagnostic and/or treatment purposes are eligible. As patients receiving less than 100 mL of iodinated contrast media may not have an increased risk of contrast-induced kidney injury, an expected use of at least 100 mL was used as inclusion criterion [3,29].

### Inclusion criteria

1) Patients undergoing an interventional or diagnostic radiological procedure in which they receive an expected >100 mL intravascular contrast including:

••Thoracic and/or abdominal endovascular aortic repair

••Endovascular aortic repair

••Digital subtraction angiography

••Percutaneous transluminal angioplasty

••Percutaneous intentional extraluminal revascularization

••Carotic artery stenting

••Percutaneous coiling/embolization procedures

••Computed tomography

2) Patients who comply with the risk criteria for CIN according to the Dutch guidelines [14]

••Peripheral vascular disease, heart failure, >75 years, anemia (Ht < 0.39 men and <0.36 women, dehydration, diuretics and/or NSAID use)

3) Written informed consent.

### Exclusion criteria

•Age <18 years

•Hemodialysis or peritoneal dialysis

•Simultaneous participation in another interventional study

•Percutaneous coiling/embolization procedures of the kidney

•Impossibility to perform RIPC, due to pathology of both arms (for example, dystrophy, recent trauma, chronic wounds)

### Study protocol

All participating patients will receive the standard hydration schedule consisting of an infusion with saline 0.9% solution 3 to 4 mL/kg/h for 4 h prior to and 4 h after contrast administration. In patients with congestive heart failure or MDRD <30 mL/min/1.73 m^2^ a long schedule is used with an infusion of saline 0.9% solution 1 mL/kg/h for 12 h prior to and 12 h after the contrast administration. Nephrotoxic drugs (for example, metformin and diuretics) are discontinued at least 24 h before and after contrast administration [14]. Patients in the experimental group of the study will receive RIPC by four cycles of ischemia and reperfusion of the forearm by inflating a blood pressure cuff around the upper arm at 50 mmHg above the actual systolic pressure during 5 min followed by 5 min of reperfusion. In the control group, patients receive sham preconditioning by inflating the blood pressure cuff to 10 mmHg below the actual diastolic pressure during 5 min followed by 5 min of reperfusion (four cycles). The time between the last inflation cycle and the start of the intervention is planned within 45 min. In the interest of blinding, the investigator ensures that the inflation pressure is not visible for both the patient and the (interventional) radiologist. All patients receive Xenetrix 300 (0.6 to 0.85 Osmol/kg H_2_O), a low osmolar, non-ionic, and hydrophilic contrast medium [30,31]. Patients will complete a questionnaire to obtain all relevant baseline characteristics such as age, weight, previous contrast procedures, diabetes, vascular-related diseases, and (discontinuation of) medication. Chart review will be performed to complement and double check this information. Blood and urine samples are taken at baseline and 4 to 6 h after contrast administration. A final blood sample is taken 48 to 72 h after contrast administration. According to the Dutch guidelines, monitoring of renal function in high-risk patients is recommended within 48 to 72 h after contrast administration. All samples will be number coded before analysis to ensure blinding of the independent investigator performing the analyses.

### Primary endpoint

The primary endpoint is change in serum creatinine form baseline to serum creatinine within 48 to 72 h after contrast administration.

### Secondary endpoint

The secondary endpoints are the incidence of CIN (defined as an absolute rise of ≥0.5 mg/dL or a relative increase of ≥25% in serum creatinine over baseline within 48 to 72 h after contrast administration), rehospitalization, hemodialysis, and mortality within 6 weeks after contrast administration.

### Ethics, informed consent

An independent ethics committee, the Central Committee on Research involving Human Subjects, Arnhem-Nijmegen, approved the protocol. Oral and written informed consent from the patient will be obtained prior to inclusion.

### Adverse events

Although RIPC by repeated insufflations of a blood pressure cuff around the upper arm is considered safe, serious adverse events possibly related to the application of RIPC will be reported to the ethical committee. Mild adverse events are: transient discomfort due to compression and/or ischemia and the formation of ecchymosis (upper arm) or petechia (lower arm).

### Power analysis

In this randomized study, the change of serum creatinine from baseline to 48 to 72 h after contrast administration will be compared between the experimental and control group. Using serum creatinine change as continuous response variable increases the power of the study. In a previous retrospective cohort study at our center including 2,169 patients at risk for contrast-induced nephropathy, serum creatinine values decreased from 120 μmol/L at baseline to 118 μmol/L at 48 to 72 h after contrast administration due to adequate hydration protocols [17]. This mean change in serum creatinine (-2 μmol/L) was normally distributed with a standard deviation of 23 μmol/L. Based on existing evidence we assume that RIPC with hydration may provide a further decrease in mean serum creatinine from baseline to 48 to 72 h of approximately 14 μmol/L as compared to hydration only. This corresponds with approximately 60% of the effect that was found by Er et al. [28]. If the true difference in the experimental and control means is 14 μmol/L, we will need to study 34 experimental and 34 controls to be able to reject the null hypothesis with a power of 0.80 and an alpha of 0.05 calculated with a one-sided independent t-test. Based on existing animal [25] and human studies [32,33] investigating the influence of RIPC on renal ischemia-reperfusion injury, we assume that RIPC does not negatively affect renal function. Therefore, one-sided testing would be appropriate for this study. Expected lost to follow-up (for example, blood sampling not realized between 48 to 72 h) is approximately 5%. For this reason 38 patients will be included in both the experimental and control arm.

### Statistical analysis

The analysis will be performed on the basis of intention-to-treat principles. Student’s t-test will be used to compare normally distributed variables, and Mann-Whitney U test will be used to compare not-normally distributed continuous data. Categorical variables will be compared with the chi-square test. If univariable analysis reveals a significant difference in baseline characteristics, then a multivariable linear regression analysis will be used to assess its impact on the primary outcome measure (that is, change in serum creatinine between baseline and 48 to 72 h after contrast administration). A subgroup analysis will be performed to assess whether the impact of RIPC on the primary outcome measure is affected by the Mehran risk score. For this analysis patients will be divided into three equal groups (that is, tertiles) according to their Mehran risk score. Statistical analyses will be performed with SPSS 20.0. A probability value of <0.05 is considered to indicate statistical significance and 95% confidence intervals will be calculated.

The RIPCIN study is registered at: http://www.controlled-trials.com/ISRCTN76496973.

## Discussion

In this study, we hypothesize that RIPC reduces the occurrence of CIN in patients at risk of acute kidney injury due to the use of contrast media. A recent randomized pilot study suggested that RIPC reduced contrast-induced kidney injury, however this study was performed in patients with an unusual high risk of CIN. A comment on this study by Mehta Oza et al. clarified that based on the reported Mehran risk score the incidence of CIN should lie between 26% and 30% instead of 40% as reported by Er et al. [34]. The authors stated that this high incidence of CIN could be attributed to a high prevalence of heart failure and diabetes mellitus in their cohort. However, if standard measures to prevent CIN, that is, hydration with saline and discontinuation of nephrotoxic drugs, were not carried out appropriately, then the incidence of CIN would also be increased. As compliance to standard preventive measures against CIN was not described by Er et al. their results do not fully justify the conclusion that RIPC, as an adjunct to standard preventive measures, effectively reduces CIN. Another important issue to address is the fact that the incidence of CIN varies with the criteria used [35]. Er et al. defined CIN as an absolute or relative increase in serum creatinine, whereas some evidence exists that both an absolute and a relative increase in serum creatinine more accurately predicts adverse events after coronary angioplasty. To overcome the flaws related to the use of different definitions of CIN, we will use the change in serum creatinine from baseline to 48 to 72 h after contrast administration as that primary endpoint in the proposed trial. As serum creatinine levels generally peak between 48 and 72 h after contrast administration, it would be ideal to measure serum creatinine at both 48 and 72 h. However, this would not be in line with Dutch and international guidelines which recommend checking renal function once between 48 and 72 h after contrast administration. In practice most patients are discharged within 24 h after contrast administration and for many it is already difficult to realize one blood sample between 48 and 72 h after contrast administration. In our view, it is appropriate for proof-of-concept studies investigating new strategies to reduce contrast-induced kidney injury to use the change in serum creatinine from baseline to 48 to 72 h as the primary endpoint. Once the efficacy of a new strategy against contrast-induced kidney injury has been confirmed, much larger clinical trials should be conducted with adverse effects after the use of contrast-media (for example, dialysis and/or death) as the primary endpoint.

## Trial status

The trial is ongoing. Currently 61 patients have been included.

## Abbreviations

CIN: Contrast-induced nephropathy; MDRD: Mean glomerular filtration rate; RIPC: Remote ischemic preconditioning.

## Competing interests

The authors of this manuscript have no competing interests to disclose.

## Authors’ contributions

TS contributed to data collection and analysis, manuscript writing, and final approval of the manuscript. TM and YW contributed to data collection, critical revision, and final approval of the manuscript. RD contributed to analysis, critical revision, and final approval of the manuscript. KW, SL and DV contributed to data collection, critical revision, and final approval of the manuscript. JW contributed to design, interpretation of the data, critical revision, and final approval of the manuscript. LS contributed to conception and design, critical revision, and final approval of the manuscript. MW contributed to conception and design, data collection, interpretation of the data, financial support, analysis, manuscript writing, and final approval of the manuscript. All authors read and approved the final manuscript.
